# Supplementary material for: Molecular insights into nucleocapsid assembly and transport in Marburg and Ebola viruses
Source: mBio. 2025 Sep 22;16(11):e01557-25. doi: 10.1128/mbio.01557-25 (PMC12607913; doi:10.1128/mbio.01557-25)
Supplement: Supplemental Figures — Figures S1 to S6. [file mbio.01557-25-s0001.pdf]

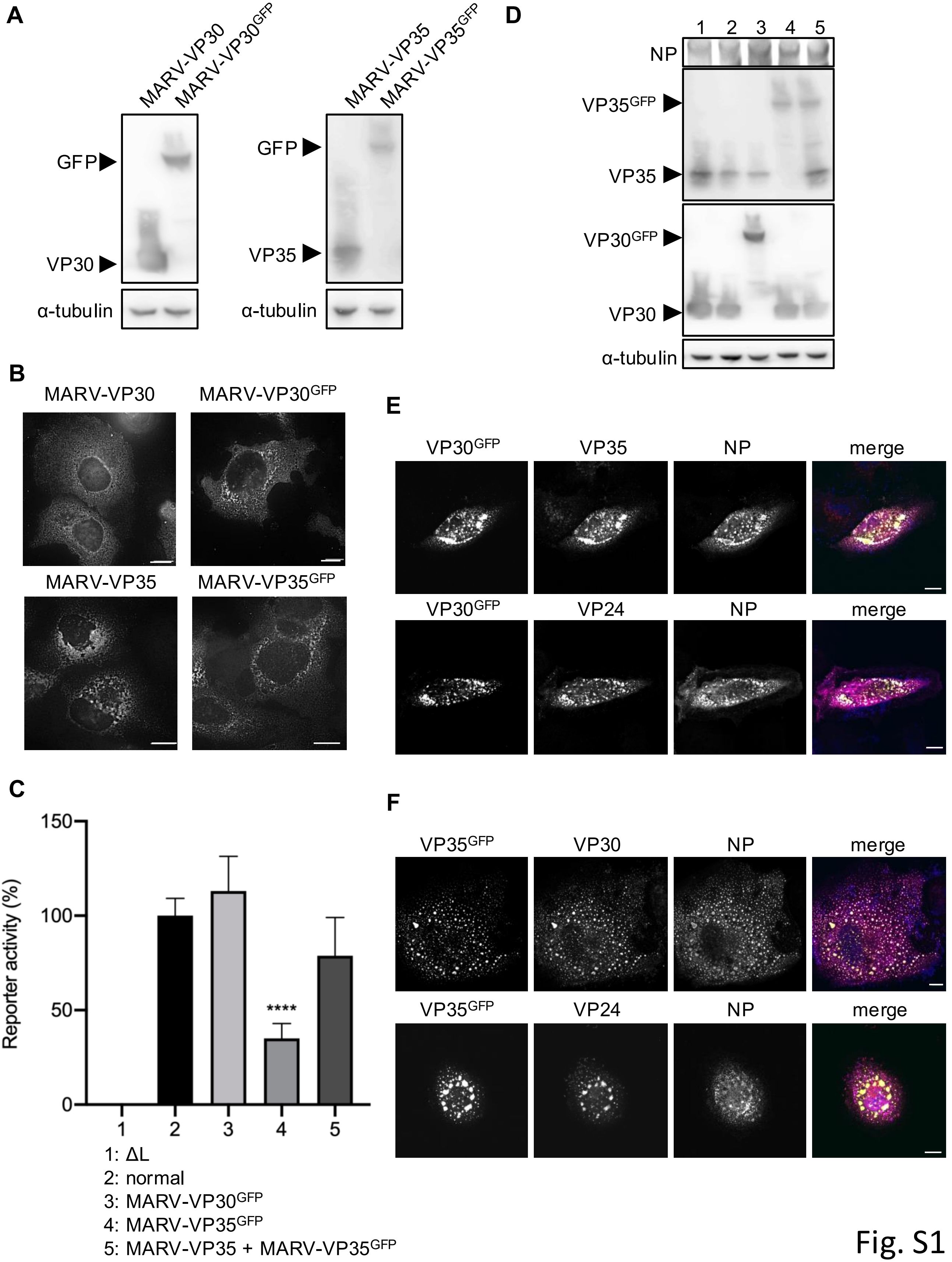
A
MARV-VP30
MARV-VP30GFP
GFP
VP30
α-tubulin
MARV-VP35
MARV-VP35GFP
GFP
VP35
α-tubulin
B
MARV-VP30
MARV-VP30GFP
MARV-VP35
MARV-VP35GFP
C
Reporter activity (%)
150
100
50
0
****
1
2
3
4
5
1: ΔL
2: normal
3: MARV-VP30GFP
4: MARV-VP35GFP
5: MARV-VP35 + MARV-VP35GFP
D
1 2 3 4 5
NP
VP35GFP
VP35
VP30GFP
VP30
α-tubulin
E
VP30GFP
VP35
NP
merge
VP30GFP
VP24
NP
merge
F
VP35GFP
VP30
NP
merge
VP35GFP
VP24
NP
merge

Fig. S1

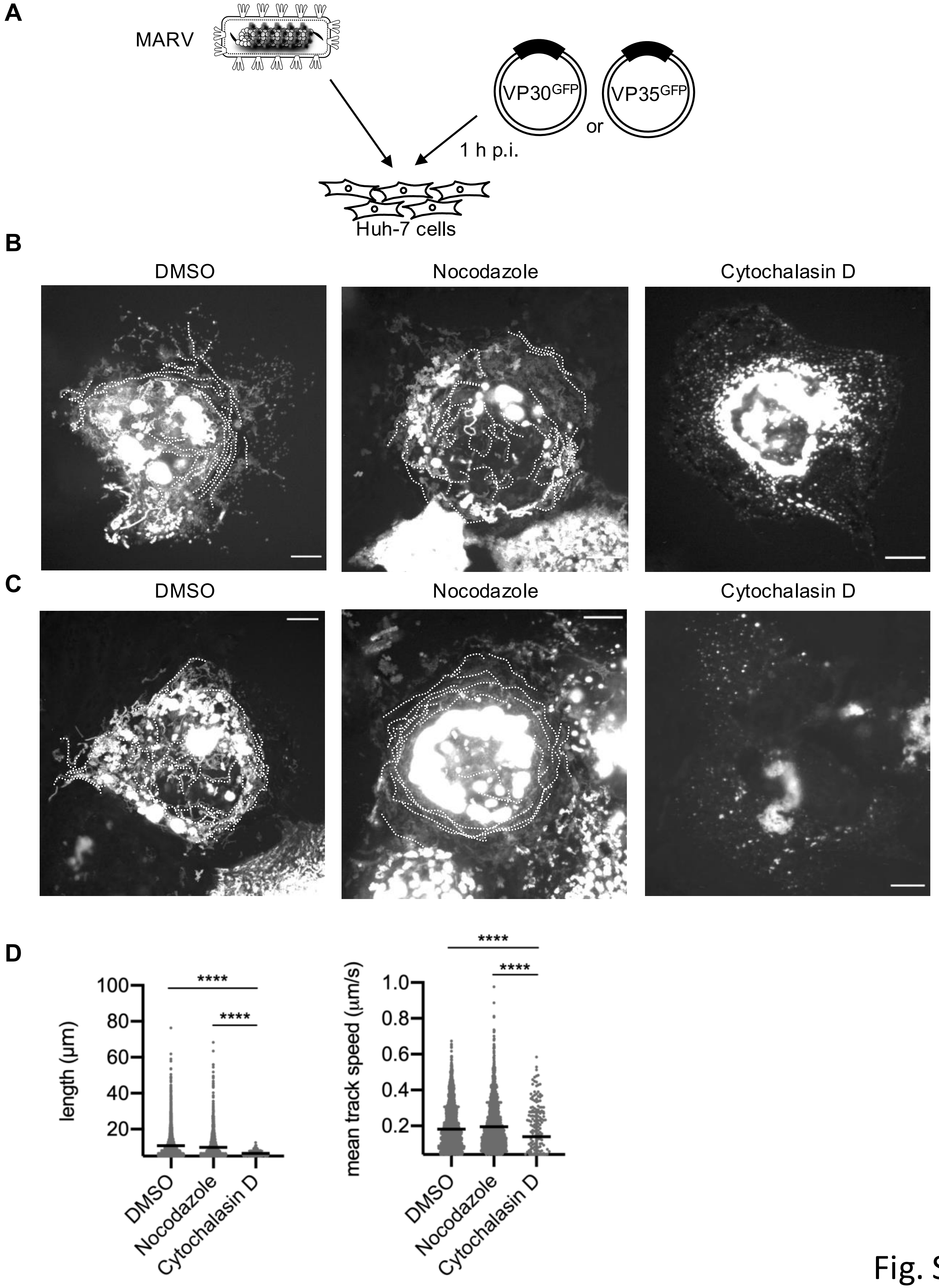
A
MARV
VP30GFP
VP35GFP
or
1 h p.i.
Huh-7 cells
B
DMSO
Nocodazole
Cytochalasin D
C
DMSO
Nocodazole
Cytochalasin D
D
length (µm)
100
80
60
40
20
****
****
DMSO
Nocodazole
Cytochalasin D
mean track speed (µm/s)
1.0
0.8
0.6
0.4
0.2
****
****
DMSO
Nocodazole
Cytochalasin D

Fig. S2

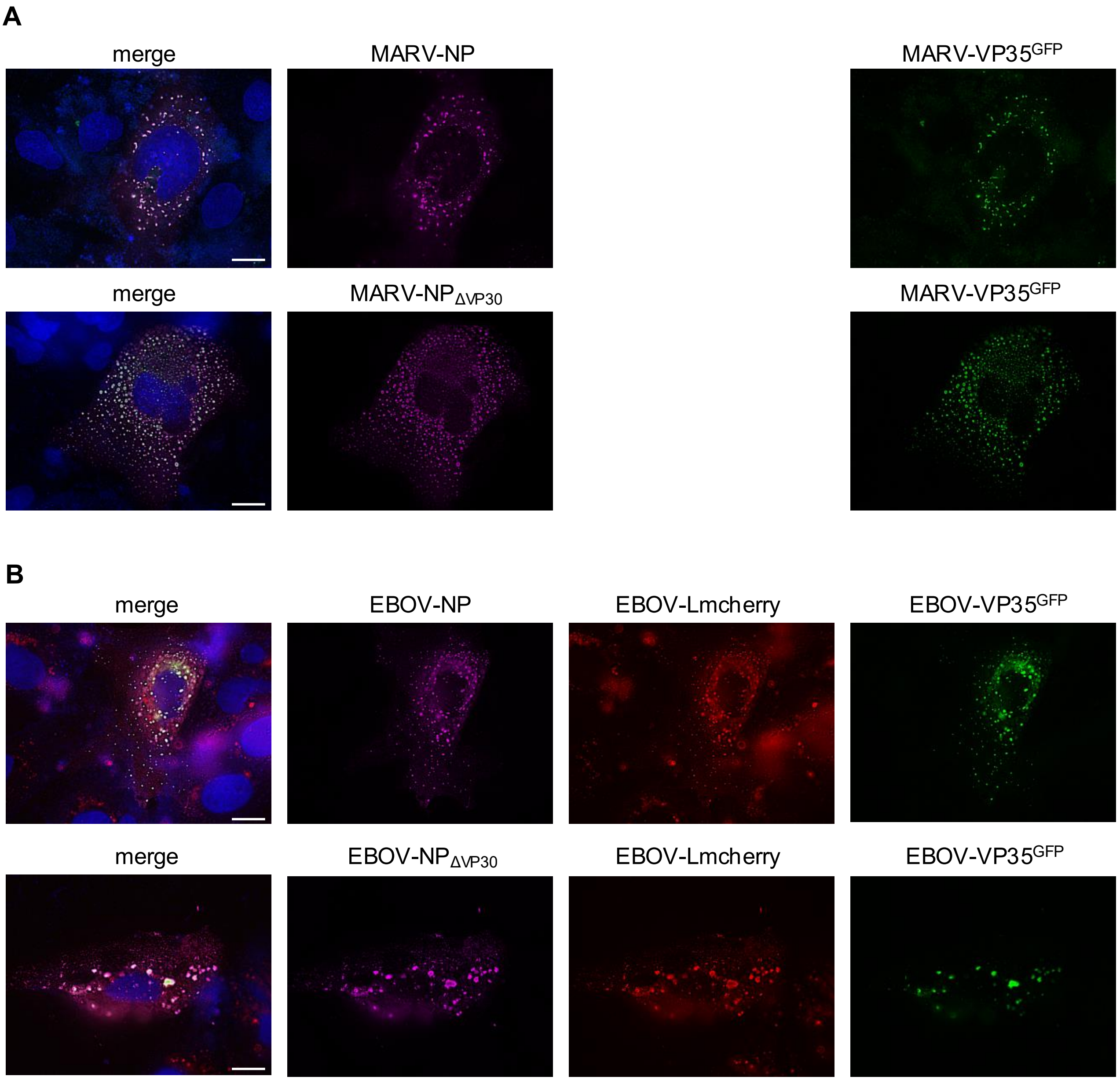
A
merge
MARV-NP
MARV-VP35GFP
merge
MARV-NPΔVP30
MARV-VP35GFP
B
merge
EBOV-NP
EBOV-Lmcherry
EBOV-VP35GFP
merge
EBOV-NPΔVP30
EBOV-Lmcherry
EBOV-VP35GFP

Fig. S3

A

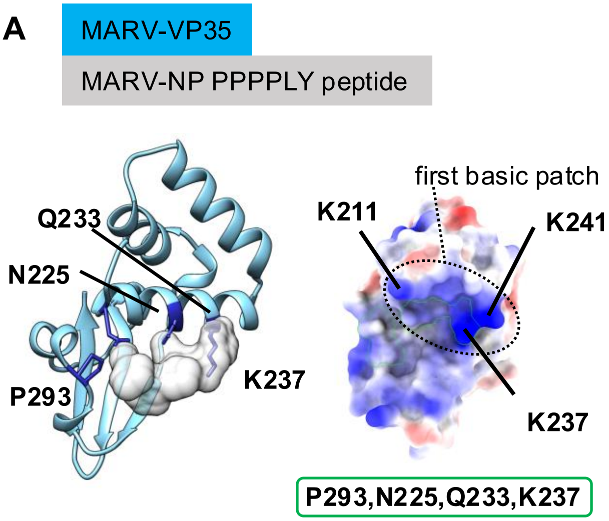

B

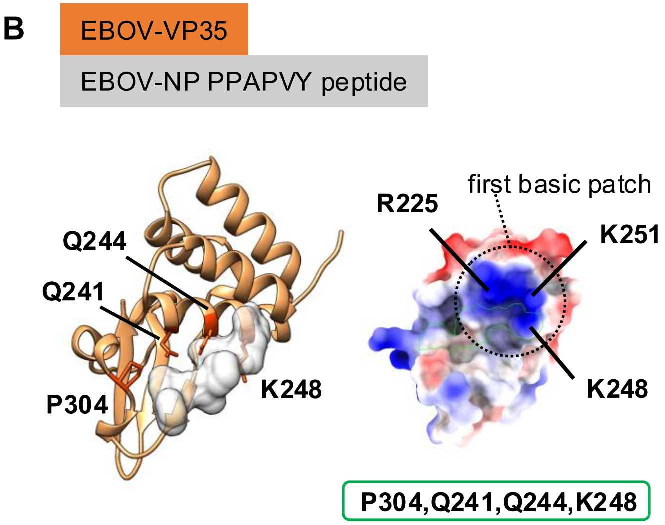

C

MARV NP peptide – VP35/VP24 interaction

interaction	prediction
wt: PPPPLY peptide – VP35	○
AAPALA peptide –VP35	1
wt: PPPPLY peptide – VP24	1
AAPALA peptide –VP24	1

EBOV NP peptide – VP35/VP24 interaction

interaction	prediction
wt: PPAPVY peptide – VP35	○
AAAAVA peptide –VP35	1
wt: PPAPVY peptide – VP24	2
AAAAVA peptide –VP24	2

1: Reliable complex structure was not predicted.
2: Both the wild type and the mutant were predicted to have the similar-binding structure (D).

D

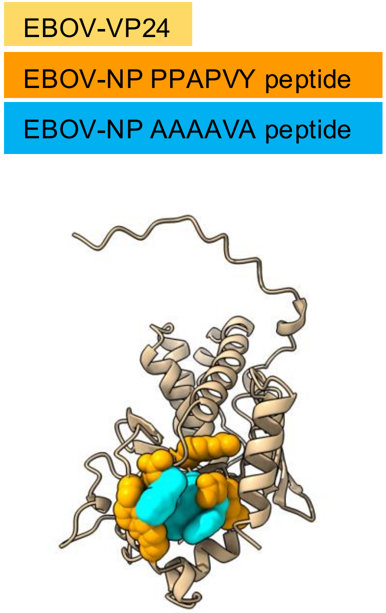

Fig. S4

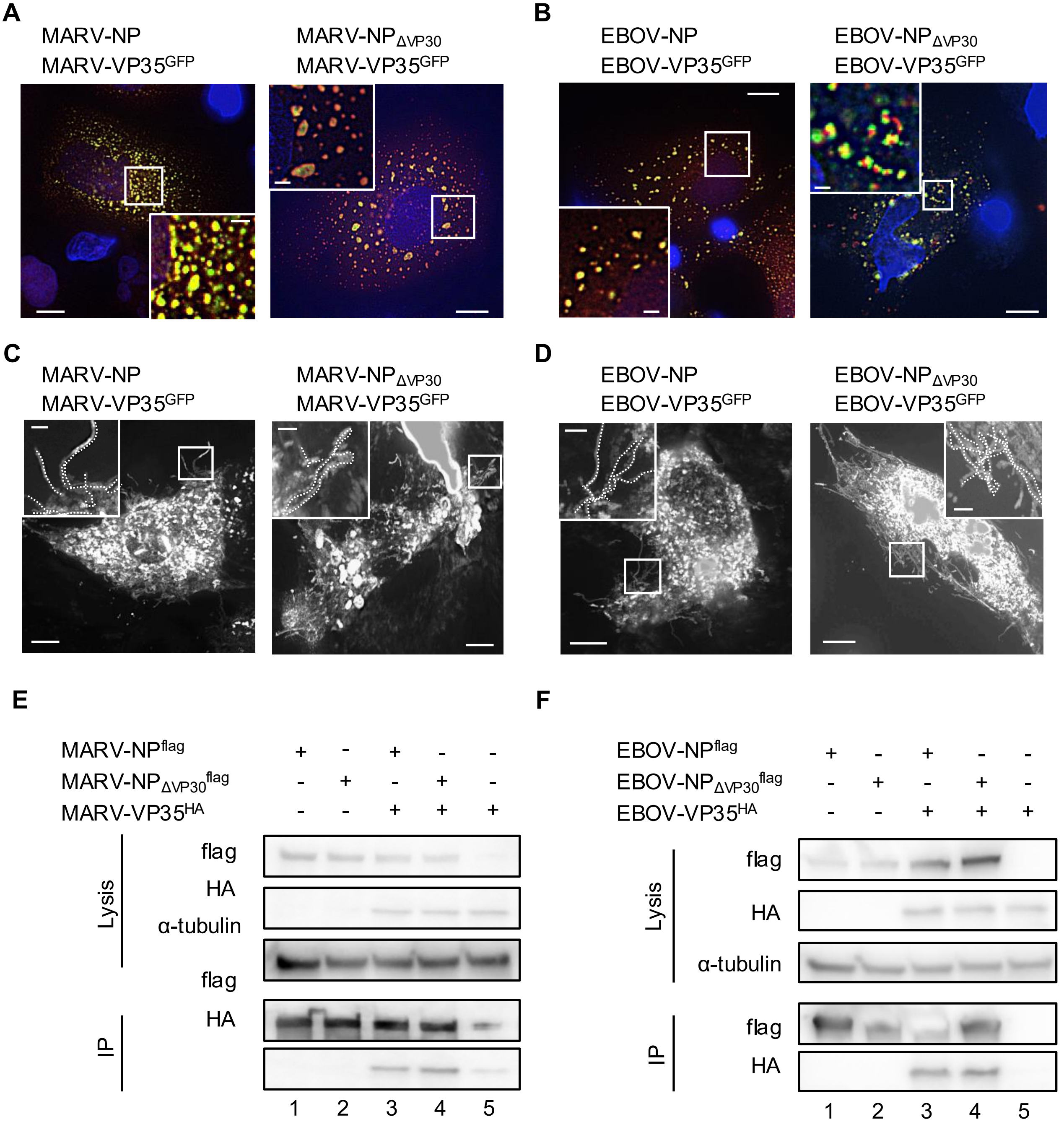
A
MARV-NP
MARV-VP35GFP
MARV-NPΔVP30
MARV-VP35GFP
B
EBOV-NP
EBOV-VP35GFP
EBOV-NPΔVP30
EBOV-VP35GFP
C
MARV-NP
MARV-VP35GFP
MARV-NPΔVP30
MARV-VP35GFP
D
EBOV-NP
EBOV-VP35GFP
EBOV-NPΔVP30
EBOV-VP35GFP
E
MARV-NPflag
MARV-NPΔVP30flag
MARV-VP35HA
Lysis
flag
HA
α-tubulin
flag
HA
IP
1 2 3 4 5
F
EBOV-NPflag
EBOV-NPΔVP30flag
EBOV-VP35HA
Lysis
flag
HA
α-tubulin
IP
flag
HA
1 2 3 4 5

Fig. S5

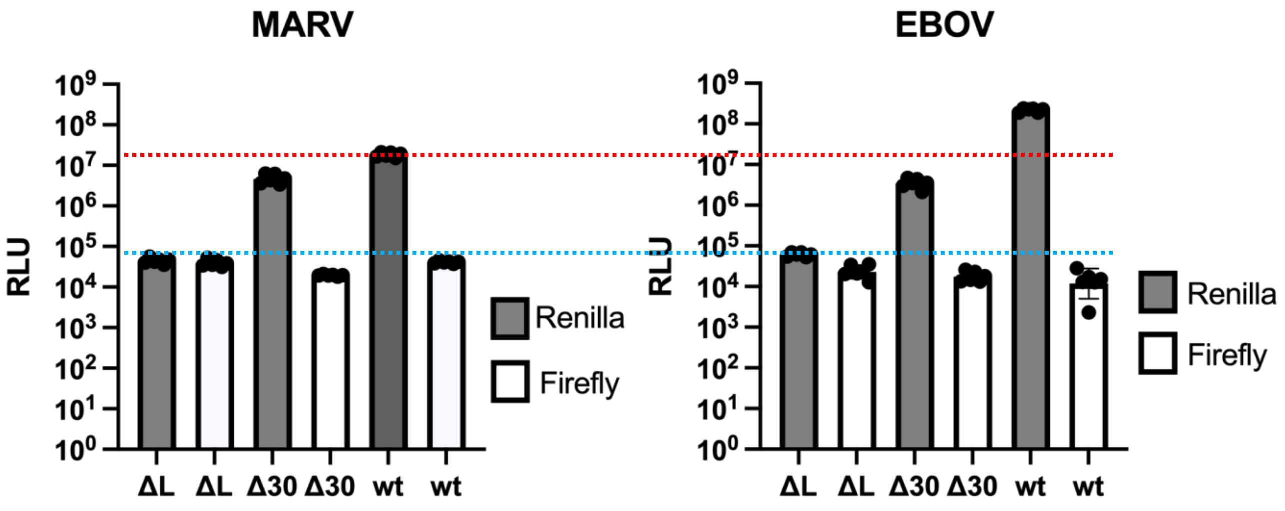
MARV
EBOV
RLU
10^9
10^8
10^7
10^6
10^5
10^4
10^3
10^2
10^1
10^0
ΔL
ΔL
Δ30
Δ30
wt
wt
Renilla
Firefly

Fig. S6
