# Supplementary material for: Molecular insights into nucleocapsid assembly and transport in Marburg and Ebola viruses
Source: mBio. 2025 Sep 22;16(11):e01557-25. doi: 10.1128/mbio.01557-25 (PMC12607913; doi:10.1128/mbio.01557-25)
Supplement: Legends — to supplemental figures and movies. [file mbio.01557-25-s0002.pdf]

Supplementary files

Figure S1. Expression and validation of GFP-conjugated VP30 and VP35 constructs. (A) HEK293 cells were transfected with the plasmids encoding MARV-VP30, MARV-VP30^{GFP} (left) as well as MARV-VP35 and MARV-VP35^{GFP} (right). Cells were harvested at 24 h p.t., lysed, and subjected to western blotting using antibodies against MARV-VP30, MARV-VP35 and α-tubulin. Western blotting confirmed similar levels of expression of MARV-VP30, MARV-VP30^{GFP}, MARV-VP35 and MARV-VP35^{GFP}. (B) Huh-7 cells were transfected with the indicated protein-coding plasmids, and their intracellular distribution was analyzed using antibodies against MARV-VP30, MARV-VP35, or autofluorescence by confocal immunofluorescence microscopy. Both non-tagged and GFP-conjugated VP30 constructs exhibited a similar diffuse distribution in the cytoplasmic region, and both non-tagged and GFP-conjugated VP35 constructs underwent perinuclear accumulation together with diffuse cytoplasmic distribution. (C) HEK293 cells were transfected with minigenome assay components. At 48 h p.t., cells were lysed, and reporter activity was measured. Lane 1: in the absence of L (negative control), lane 2: normal minigenome

components, lane 3: MARV-VP30^{GFP} instead of MARV-VP30, lane 4: MARV-VP35^{GFP} instead of MARV-VP35, lane 5: MARV-VP35 and MARV-VP35^{GFP} -expressing cells. The value of MARV-VP30 and MARV-VP35 -expressing cells (lane 2) was set to 100%. The mean and SD of three independent experiments are shown. Asterisks indicate statistical significance; ***P < 0.001. MARV-VP30^{GFP} demonstrated a similar level of reporter activity as that of the wild-type (lane 3), whereas MARV-VP35^{GFP} was not as functional as the respective wild-type (lane 4). However, supplementary expression of MARV-VP35 rescued minigenome activity in the presence of MARV-VP35^{GFP} (lane 5). Protein expression in the minigenome assay was confirmed by western blotting. (D) Western blot analysis of cells after minigenome assays using antibodies against MARV-NP, MARV-VP30, MARV-VP35 and α-tubulin. (E) Huh-7 cells transfected with MARV-viral protein-encoding plasmids for NP, VP35, VP24, and VP30^{GFP} were fixed at 24 h p.t. and analyzed by confocal immunofluorescence microscopy. Intracellular distributions of each viral protein were analyzed using specific antibodies (MARV-NP, MARV-VP35, MARV-VP24). (F) Huh-7 cells transfected with plasmids encoding NP, VP35, VP35^{GFP}, VP24, and VP30 were fixed at 24 h

p.t. and analyzed by confocal immunofluorescence microscopy. Intracellular distributions of each viral protein were analyzed using specific antibodies (MARV-NP, MARV-VP30, MARV-VP24). Confocal microscopic analyses demonstrated colocalization of MARV proteins VP30^{GFP}, NP, VP35, and VP24 inside inclusion bodies, the sites where NCLSs were formed, and in small punctuate, dot-like structures predominately visible at the cell periphery, representing NCLSs (Fig. 1E). Colocalization of MARV proteins VP35^{GFP}, NP, VP30, and VP24 was also observed in the inclusion bodies and small dot-like structures (Fig. 1F). Altogether, these results indicate that the fluorescent fusion nucleocapsid proteins MARV-VP30^{GFP} and MARV-VP35^{GFP} were functional in reporter assays, and were recruited into inclusion bodies and incorporated into individual NCLSs.

Figure S2. Live-cell imaging analysis of MARV nucleocapsid transport. (A) Schematic representation of experimental settings for nucleocapsid transport in MARV-infected cells. Huh-7 cells were infected with MARV at an MOI of 1, and then transfected with plasmids encoding VP30GFP or VP35GFP at 1 h p.i. Live-cell imaging microscopy was conducted in a biosafety level-4 (BSL-4) facility. (B, C) Live-cell imaging analysis of VP30^{GFP} (B) or VP35^{GFP} (C) -labeled

nucleocapsids was performed at 20 h p.t. At 17 h p.t., different cytoskeleton-modulating drugs were added to the culture medium: 0.15% DMSO, 15 μM nocodazole, or 0.3 μM cytochalasin D, and the cells were incubated for an additional 3 h. Long lines reflecting directional nucleocapsid transport over long distances were detected in maximum intensity projections of time-lapse images. No differences in the characteristics of nucleocapsid transport were observed in MARV-VP30^{GFP}- or MARV-VP35^{GFP} -expressing cells in accord with previous reports (1, 2). Next, we analyzed the transport characteristics of MARV nucleocapsids with the actin depolymerizing drug cytochalasin D, or the microtubule-depolymerizing drug Nocodazole-treated cells. The dotted lines indicate typical NCLS trajectories. (D) The detected signals in VP30^{GFP}-labelled NCLS were analyzed using the Imaris software. The length of nucleocapsid trajectories and velocity of nucleocapsid transport were evaluated. The y-axis represents signal counts. Lines indicate the mean ± SD. Asterisks indicate statistical significance; ****P* < 0.0001, *****P* < 0.00001. Images show the maximum intensity projection of time-lapse images of cells recorded for approximately 5 min; images were acquired every 3 s. Scale bars: 10 μm.

Nocodazole treatment did not alter the trajectory, length, or velocity of nucleocapsid transport compared to those of the control (dimethyl sulfoxide: DMSO), whereas cytochalasin D treatment induced immediate cessation of long-distance transport, indicating that nucleocapsid transport is dependent on actin polymerization.

Figure S3. Recruitment of L and VP35 to inclusion bodies formed by PPxPxY motif variants of NP

(A) Huh-7 cells were transfected with plasmids encoding MARV-VP35GFP, MARV-L, along with either MARV-NP or MARV-NP$_{\Delta VP30}$. At 48 hours post-transfection, the cells were fixed and visualized using GFP and anti-NP antibody. VP35 was accumulated in both NP-forming inclusions.

(B) Huh-7 cells were transfected with plasmids encoding EBOV-VP35GFP, EBOV-L, along with either EBOV-NP or EBOV-NP$_{\Delta VP30}$. At 48 hours post-transfection, the cells were fixed and visualized using GFP, mCherry and anti-NP antibody. L and VP35 were accumulated in both NP-forming inclusions.

Scale bars: 10 μm.

Figure S4. AlphaFold 2/3 structural predictions of VP35/VP24-PPxPxY

binding models and NP-VP35 interactions.

(A, B) Representative predicted structures of MARV-PPPPLY and MARV-VP35 complexes and EBOV-PPAPVY and EBOV-VP35 complexes. Only reliably predicted C-terminal globular domains in MARV VP35 (amino acid residues 217-340) (pIDDT score >70) were shown with the interacting PPAPVY peptide (white, surface representation). (B) The predicted EBOV VP35- PPAPVY structures were superimposed in advance to be presented in the same orientation as (A). Using Chimera X software, we visualized the electrostatic potential of VP35 protein and indicated the relative positions of the basic amino acids that form the first basic patch as well as the interaction sites of the PPxPxY motif (shown in the green frame). MARV-VP35 was analyzed using PDB ID: 4gh9 (A, right), while EBOV-VP35 was analyzed using PDB ID: 3fke (B, right) (C) The predicted structures of peptide-protein complexes are summarized in the table. (D) Representative predicted structures of EBOV-PPAPVY and EBOV-VP24 complexes and EBOV-AAAAVA and EBOV-VP35 complexes.

Figure S5. NP-VP35 proteins interaction was not regulated by PPxPxY motif.

(A, B) Immunofluorescence assay in Huh-7 cells. Cells were transfected with the

indicated protein-encoding plasmids (A: MARV, B: EBOV). Intracellular distribution of proteins noted above the images was visualized using NP-specific antibodies and autofluorescence, and merged images were visualized. The small, boxed areas are enlarged at the four corners. Scale bars: 10 μm (Scale bar in *Insets*, 2 μm). (C,D) Live-cell imaging in Huh-7 cells. Cells were transfected with the indicated protein -encoding plasmids together with MARV-VP35 and MARV-VP24-encoding plasmids (C). The cells were transfected with indicated proteins -encoding plasmids together with EBOV-VP35 and EBOV-VP24-encoding plasmids (D). Live-cell imaging analysis was started from 20 h p.t. The image shows the maximum-intensity projection of time-lapse images of cells, recorded for 2-3 min; images were captured every 2–3 s. The small boxed areas are enlarged at the four corners. Scale bars: 10 μm (Scale bar in *Insets*, 2 μm). The dotted lines indicate typical NCLS trajectories. (E,F) Immunoprecipitation assays in HEK293 cells. Cells were transfected with the indicated protein-encoding plasmids (E: MARV, F: EBOV). NP-encoding plasmids were fused with a FLAG tag, cells were lysed, and protein complexes were precipitated using mouse anti-FLAG M2 agarose at 48 h p.t. An aliquot of cell lysate (input) was collected before

precipitation. Elution was achieved using SDS sample buffer. Western blot analysis was performed using FLAG-, VP35-, and α tubulin-specific antibodies.

Figure S6. Differences between MARV and EBOV minigenome assays based on actual values.

(A) HEK293 cells were transfected with MARV minigenome assay components. (B) HEK293 cells were transfected with EBOV minigenome assay components. At 48 hours post-transfection, cells were lysed, and reporter activity was measured. The activity levels of cells transfected with the respective wild-type minigenome components (A and B) are compared to the negative control (absence of L expression, ΔL), which represents the background activity (blue dotted line). Although there was no difference in the measured ΔL values between MARV and EBOV, the wild-type activity values were approximately 10 times higher for EBOV than for MARV (red dotted line), indicating a difference in sensitivity.

Movie S1. Transport of nucleocapsids labeled with VP30$^{GFP.}$ Huh-7 cells expressing VP30^{GFP} were infected with MARV. The cells were treated at 17 h p.t. with 0.15 μM nocodazole for 3 h and analyzed by time-lapse microscopy. Time-

lapse images of cells were recorded for 5 min, and images were captured every 2 s.

Movie S2. Transport of nucleocapsids labeled with VP35^{GFP}. Huh-7 cells expressing VP35^{GFP} were infected with MARV. The cells were treated at 17 h p.t. with 0.15 µM nocodazole for 3 h and analyzed using time-lapse microscopy. Time-lapse images of cells were recorded for 6 min, and images were captured every 2 s.

Movie S3. Huh-7 cells were transfected with plasmids encoding MARV-NP, MARV-VP35, MARV-VP24, and MARV-VP35^{GFP}. The cells were treated at 17 h p.t. with 0.15 µM nocodazole for 3 h. Time-lapse images of the cells were recorded for 90 s, and images were captured every 2 s.

Movie S4. Huh-7 cells were transfected with plasmids encoding MARV-NP, MARV-VP35, MARV-VP24, and MARV-VP35^{GFP}. The cells were treated at 17 h p.t. with 0.3 µM cytochalasin D. Time-lapse images of the cells were recorded for 90 s, and images were captured every 2 s.

Movie S5. Huh-7 cells were transfected with plasmids encoding MARV-NP, MARV-VP35, MARV-VP24, and EBOV-VP30^{GFP}. Time-lapse images of the cells were recorded for 90 s, and images were captured every 2 s.

Movie S6. Huh-7 cells were transfected with plasmids encoding EBOV-NP, EBOV-VP35, EBOV-VP24, and MARV-VP30^{GFP}. Time-lapse images of the cells were recorded for 90 s, and images were captured every 2 s.

References

1. Schudt G, Kolesnikova L, Dolnik O, Sodeik B, Becker S. 2013. Live-cell imaging of Marburg virus-infected cells uncovers actin-dependent transport of nucleocapsids over long distances. Proc Natl Acad Sci U S A 110:14402-7.
2. Dolnik O, Kolesnikova L, Welsch S, Strecker T, Schudt G, Becker S. 2014. Interaction with Tsg101 is necessary for the efficient transport and release of nucleocapsids in marburg virus-infected cells. PLoS Pathog 10:e1004463.
